# Advanced machine learning for predicting individual risk of flares in rheumatoid arthritis patients tapering biologic drugs

**DOI:** 10.1186/s13075-021-02439-5

**Published:** 2021-02-27

**Authors:** Asmir Vodencarevic, Koray Tascilar, Fabian Hartmann, Michaela Reiser, Axel J. Hueber, Judith Haschka, Sara Bayat, Timo Meinderink, Johannes Knitza, Larissa Mendez, Melanie Hagen, Gerhard Krönke, Jürgen Rech, Bernhard Manger, Arnd Kleyer, Marcus Zimmermann-Rittereiser, Georg Schett, David Simon, on behalf of the RETRO study group

**Affiliations:** 1 Digital Health, Siemens Healthcare GmbH, 91052 Erlangen, Germany; 2 Department of Internal Medicine 3 - Rheumatology and Immunology, Friedrich-Alexander University (FAU) Erlangen-Nürnberg and Universitätsklinikum Erlangen, 91054 Erlangen, Germany; 3 Deutsches Zentrum fuer Immuntherapie (DZI), 91054 Erlangen, Germany; 4 Section Rheumatology, Sozialstiftung Bamberg, 96049 Bamberg, Germany; 5 Vinforce Study Group, St. Vincent Hospital, Medical University of Vienna, 1090 Vienna, Austria

**Keywords:** Rheumatoid arthritis, Machine learning, Flare prediction

## Abstract

**Background:**

Biological disease-modifying anti-rheumatic drugs (bDMARDs) can be tapered in some rheumatoid arthritis (RA) patients in sustained remission. The purpose of this study was to assess the feasibility of building a model to estimate the individual flare probability in RA patients tapering bDMARDs using machine learning methods.

**Methods:**

Longitudinal clinical data of RA patients on bDMARDs from a randomized controlled trial of treatment withdrawal (RETRO) were used to build a predictive model to estimate the probability of a flare. Four basic machine learning models were trained, and their predictions were additionally combined to train an ensemble learning method, a stacking meta-classifier model to predict the individual flare probability within 14 weeks after each visit. Prediction performance was estimated using nested cross-validation as the area under the receiver operating curve (AUROC). Predictor importance was estimated using the permutation importance approach.

**Results:**

Data of 135 visits from 41 patients were included. A model selection approach based on nested cross-validation was implemented to find the most suitable modeling formalism for the flare prediction task as well as the optimal model hyper-parameters. Moreover, an approach based on stacking different classifiers was successfully applied to create a powerful and flexible prediction model with the final measured AUROC of 0.81 (95%CI 0.73–0.89). The percent dose change of bDMARDs, clinical disease activity (DAS-28 ESR), disease duration, and inflammatory markers were the most important predictors of a flare.

**Conclusion:**

Machine learning methods were deemed feasible to predict flares after tapering bDMARDs in RA patients in sustained remission.

**Supplementary Information:**

The online version contains supplementary material available at 10.1186/s13075-021-02439-5.

## Introduction

Rheumatoid arthritis (RA) is the archetypal chronic inflammatory disease. While most RA patients were in an active disease state two decades ago, continuous improvements in the management of RA today allows many patients to experience low disease activity or even remission. For instance, data from the NOR-DMARD registry revealed an up to 3-fold increased chance of remission in the last 10 to 20 years [1]. At present, approximately 50% of patients with early RA reach sustained remission [2]. This improvement in outcomes is due to a number of changes in RA management, namely (i) tight disease control based on treat-to-target concept, (ii) earlier diagnosis of the disease, and (iii) an expansion of RA treatment with the use of more efficient drugs, such as biological disease modifying anti-rheumatic drugs (bDMARDs) or targeted-synthetic DMARDs (tsDMARDs) [3].

For RA patients in sustained remission, tapering of anti-rheumatic treatment has been proposed [4, 5]. Data from randomized controlled and observational studies on DMARD tapering suggested that up to 50% of patients who reach sustained remission are able to successfully taper DMARDs [3]. In case of a flare, reinitiating treatment with the withdrawn drug usually restores remission [4, 6]. Several risk factors for flares, such as ACPA positivity or synovitis detected by ultrasound [4, 7, 8] have been proposed at the population level. However, the prediction of an individual patient’s flare risk upon treatment tapering remains challenging. Hence, reliable models based on machine learning (ML) algorithms could be helpful tools for individual flare prediction. ML includes a set of techniques for making successful predictions based on past experience [9]. Although there is an ongoing development of methods for statistical learning starting from 1960s, this field had a rapid and impressive surge thanks to the substantial increase in the amount of routinely collected, digitalized data, and improvements in computation power that made implementation of previously intractable methods of analysis possible.

The purpose of this study was to investigate the feasibility of building a predictive ML model using data from a clinical trial in order to estimate the individual flare probability in RA patients in persistent remission, who taper their biological DMARD treatment. To this end, we used data from the interim analysis of the REduction of Therapy in patients with rheumatoid arthritis in ongoing remission (RETRO) study [9].

## Methods

### The RETRO dataset

RETRO is an investigator-initiated multi-center, randomized controlled, open-label, parallel-group phase-III trial (EudraCT number 2009-015740-42), where RA patients in stable remission were randomized to one of 2 treatment tapering arms or a control arm and observed at regular intervals for incident flares for 12 months; a more detailed description of the study is provided in supplementary material. The preliminary results of this trial was previously published [4] and showed that tapering and stopping DMARD therapy (including conventional and biologic DMARDs) in RA patients is possible but associated with increased incidence of flares. All baseline visits of RETRO participants that used bDMARDs at study baseline and their follow-up visits that contained non-missing outcome data were eligible. Follow-up visits were excluded if it was unknown whether a flare had happened within the next 14 weeks or not (Fig. Supp. 1). This decision was made so that the prediction horizon would be exact as well as to avoid additional complexity from modeling the effects of time.

The outcome variable was a binary indicator of whether a patient suffered a flare within 14 weeks after a given visit. Remission was defined as Erythrocyte Sedimentation Rate-based Disease Activity Score in 28 joints (DAS-28 ESR) of less than 2.6 and a DAS-28 ESR value exceeding this threshold was defined as a flare. Potential predictors of flares included patient characteristics (age, gender, BMI, smoking, alcohol consumption), disease course variables including previous disease activity and functional status (tender and swollen joint counts, DAS-28 ESR, disease and remission duration, indicator of previous flares, Health Assessment Questionnaire – HAQ, patient and physician global assessment, time in study), medication data (ATC code, dose, indicators for subcutaneous-administration, co-treatment with MTX and other DMARDs), and laboratory data (C-reactive protein (CRP), erythrocyte sedimentation rate (ESR), rheumatoid factor (RF), and anti-citrullinated protein antibodies (ACPA) and Multi-Biomarker Disease Activity score [10]). Finally, a percent dose change variable indicating whether and by how much the treatment dose was changed at each observation was included.

### Data analysis

We prepared tables to describe our study sample using relevant summary statistics. Model generation included three steps. In the first step, data was prepared for modeling by selecting relevant variables and visits that fulfilled aforementioned criteria. The second and third steps, model training and testing, respectively, constituted an iterative procedure called nested cross-validation. This procedure is used to optimize algorithm parameters and to estimate the performance of the modeling approach on the new data. Nested cross-validation includes two cross-validation loops, namely an outer loop for performance estimation, and an inner loop for parameter optimization. In the outer loop, at first the data is split into the training set (80% in our case) and the test set (20%), taking care that all visits of a single patient end up in one of these sets (to avoid data leakage while measuring performance). The training set is given to the inner cross-validation where it is further split into three folds. For each combination of algorithm parameters, two of the folds in the inner loop are used to train a model and one fold for measuring its performance (validation). This is iteratively repeated three times, i.e., each time a different fold played a role of a validation set. Finally, the results obtained on the validation sets are averaged and a parameter combination which provided the highest performance in the inner loop is selected as optimal. Then, the model is trained on the 80% of the original data (i.e., the training set) using the optimal hyper-parameters selected in inner-loop model tuning and its performance is measured on the test set, which was not used so far neither for training nor for optimization. In the second iteration of the outer loop, another 20% of data is selected as a test set and the whole procedure was repeated. In the essence, this is a 5 × 3 nested cross-validation procedure, where the outer loop had 5 splits and the inner loop 3.

We undertook this 5 × 3 nested cross-validation to estimate the predictive performance of each one of 4 different basic classification methods and one stacking method. The basic classification methods were logistic regression (Fig. Supp. 2), k-nearest neighbors (Fig. Supp. 3), naïve Bayes classifier (Fig. Supp. 4), and random forests (Fig. Supp. 5) [11, 12]. The stacking method (Fig. Supp. 6, 7) was a logistic regression which used the predictions made by the aforementioned 4 basic classification methods as predictors and not the actual predictors; analogous to a dimensionality reduction procedure or propensity score method. A detailed description of the classification methods is given in the supplementary methods section.

The best performing model was selected based on the mean area under the receiver operating characteristics curve (AUC) from 5 cross-validation cycles. For each cycle, we also generated 2 × 2 contingency tables (confusion matrix) for the true vs. predicted flare status. Predicted flare status was labeled based on a predicted risk threshold selected using the Youden index. From these contingency tables, we calculated the mean sensitivity, specificity, and accuracy of the best performing model.

We presented model-diagnostics for the best performing model to assess and explain the learning process and understand the influence of individual predictors on the prediction performance. We prepared an algorithm learning curve depicting the model performance in training and cross-validation as a function of the number of visits supplied for training. We calculated relative importance of predictors during cross-validation, i.e., the relative amount of change in average predictive performance attributable to each predictor. This was accomplished by calculating the change in AUC by repeating the cross-validation testing for every predictor in each cross-validation cycle, where one predictor in the test set was randomly rearranged (permuted) at a time [12]. We tested the sensitivity of the models to missing data by recalculating model performance after randomly declaring a given proportion of data-points as missing values. Finally, we plotted the predicted risk of flares against the observed proportion in order to assess model calibration. This was achieved by grouping the data into deciles of predicted risk and plotting the mean predicted risk in each decile against the observed proportion of flares where the *y* = *x* line indicates perfect calibration.

## Results

We included 135 visits from 41 patients that were enrolled in the RETRO trial tapering bDMARDs (Fig. Supp. 1) of whom 20 patients experienced a total of 31 flares. The maximum DAS-28 observed was 4.75, the maximum tender joint count was 6, swollen joint count was 8 and ESR was 120. The mean DAS-28 was in the remission range throughout all the visits (1.87). Detailed baseline patient characteristics are presented in Table 1 and time-varying patient characteristics are presented in Table 2.

**Table 1:** Baseline patient characteristics

Patient characteristics (***n*** = 41)	
Age, years	53.3 (11.3)
Female gender, *N* (%)	24 (58.5)
Disease duration, years	10 (9)
Smoking, *N* (%)	
Current smoker	5 (12.2)
Ex-smoker	13 (31.7)
Never smoker	23 (56.1)
Remission duration, months	16.7 (14.4)
DAS-28 ESR, units	1.62 (0.68)
ESR, mm/h	11.5 (9.5)
CRP, mg/dL	0.26 (0.51)
Positive RF, *N* (%)	26 (63.4)
Positive ACPA, *N* (%)	28 (68.3)
Methotrexate use, *N* (%)	27 (65.9)
Other csDMARD use, *N* (%)	2 (4.9)
Biological DMARD use, *N* (%)	
Adalimumab	14 (34.1)
Tocilizumab	10 (24.4)
Etanercept	9 (22.0)
Certolizumab pegol	6 (14.6)
Golimumab	2 (4.9)
Patients with flare, *N* (%)	20 (48.8)

Values are means (SD) if not stated otherwise

*ACPA* anti-citrullinated peptide antibodies, *CRP* C-reactive protein, *DAS-28* Disease Activity Score 28 joints, *DMARD* disease-modifying anti-rheumatic drugs, *cs* conventional synthetic, *ESR* erythrocyte sedimentation rate

**Table 2:** Summary of time-varying characteristics over all study visits

	Mean (SD)	Range
CRP, mg/dl	0.48 (1.48)	(0.01–12.60)
ESR, mm/h	15.14 (44.05)	(1–120)
Tender joint count	0.33 (0.92)	(0–6)
Swollen joint count	0.41 (1.19)	(0–8)
Patient’s global assessment, VAS, mm	7.73 (14.57)	(0–100)
DAS28-ESR	1.87 (0.91)	(0.00–4.75)
Health Assessment Questionnaire	0.25 (0.50)	(0.00–2.88)
Multi-biomarker disease activity	23.23 (10.75)	(13–49)
Dose percentage (ratio of the full dosage)	0.67 (0.29)	(0.00–1.00)
Relative week of visit (baseline visit is week 0)	23.81 (17.53)	(0–59)
BMI, kg/m^2^	25.19 (3.86)	(17.5–39.45)
Dose percentage change*	−0.06 (0.27)	(−1.0–1.00)
Previous flare indicator (year/*n*)	0.23 (0.42)	(0.00–1.00)

*BMI* body mass index, *CRP* C-reactive protein, *DAS-28* Disease Activity Score 28 joints, *ESR* erythrocyte sedimentation rate

*Current dose percentage – previous dose percentage

**Fig. 1:**
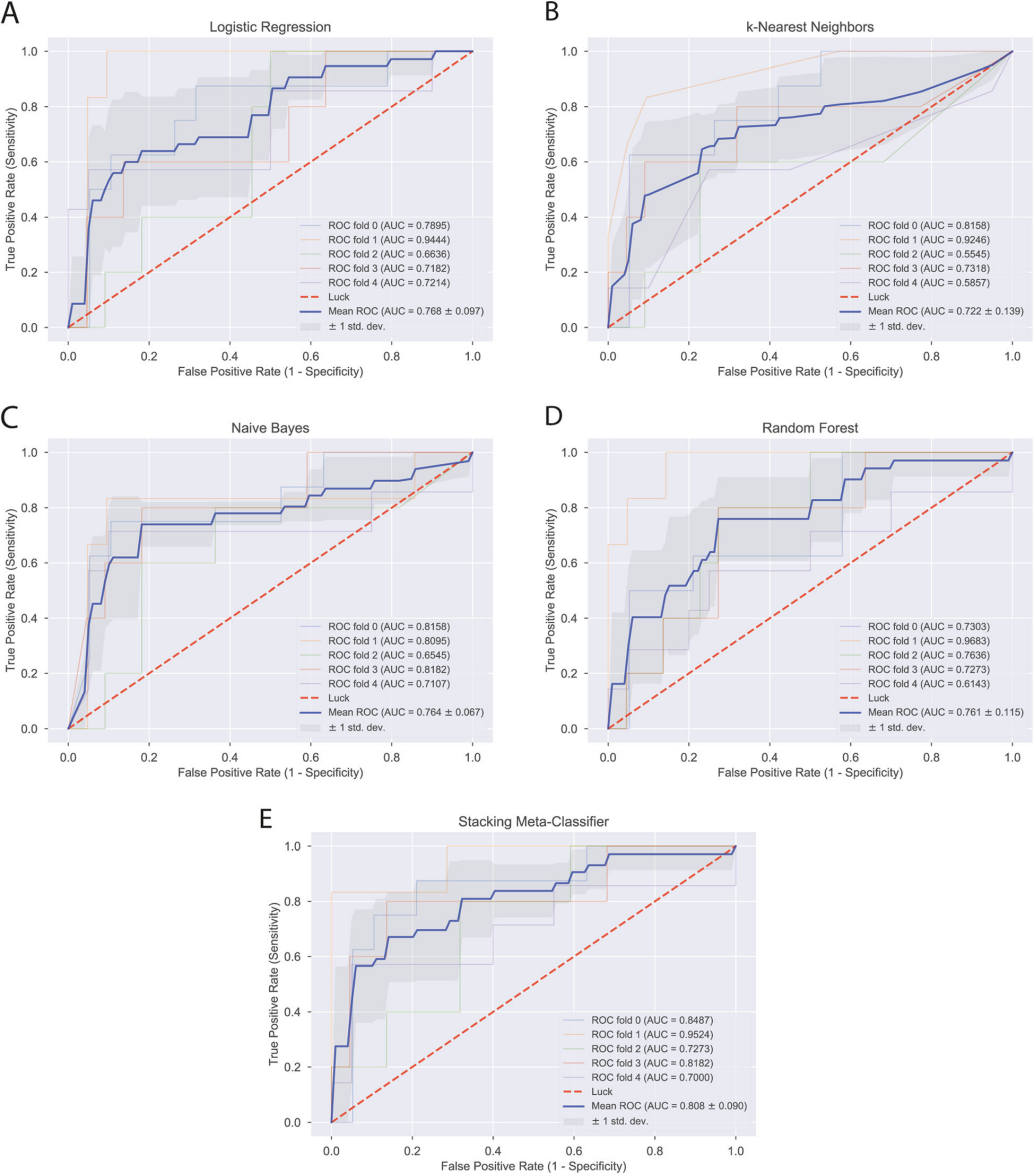
(**a**) Logistic Regression, (**b**) k-Nearest Neighbors, (**c**) Naïve Bayes, (**d**) Random Forest, (**e**) Stacking-Meta Classifier

The AUC for predicting a flare ranged from 0.72 to 0.81 using different learning methods (Fig. 1a–e). Numerically, the best performing method was the stacking meta-classifier logistic regression that predicted flares with an overall mean AUC of 0.81 (Fig. 1e). The mean (SD) specificity, sensitivity, and accuracy for this model in cross validation was 0.86 (0.11), 0.78 (0.11), and 0.81 (0.08), respectively. Contingency tables from 5 rounds of cross-validation using the stacking meta-classifier are presented in Table 3. Combining somewhat limited (e.g., piecewise linear or quadratic) decision boundaries of different models into a single powerful and flexible predictive model using the stacking approach is one of the main results of this paper.

**Table 3:** Contingency tables of true and predicted* flare status in cross-validation test folds of the stacking meta-classifier model

Fold-1		Predicted flare status	
		No	Yes
Observed flare status	No	15	4
	Yes	1	7
Fold-2		Predicted flare status	
		No	Yes
Observed flare status	No	21	0
	Yes	1	5
Fold-3		Predicted flare status	
		No	Yes
Observed flare status	No	15	7
	Yes	1	4
Fold-4		Predicted flare status	
		No	Yes
Observed flare status	No	19	3
	Yes	1	4
Fold-5		Predicted flare status	
		No	Yes
Observed flare status	No	19	1
	Yes	3	4

*Binary predictions based on predicted risk threshold selected using Youden’s index in each fold

**Fig. 2:**
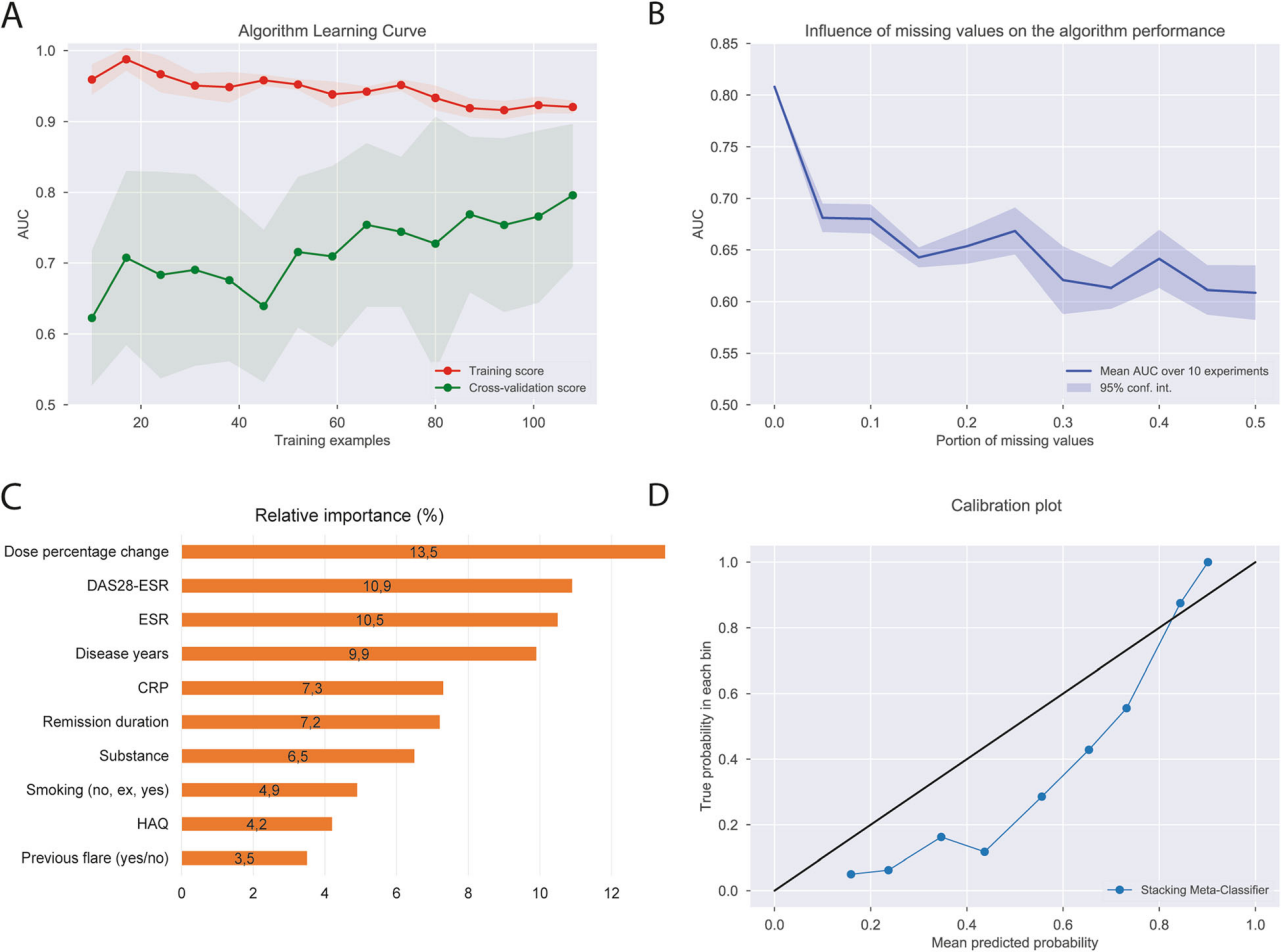
Model diagnostics. Algorithm learning curve is depicted in **a**, **b** shows influence of missing data on the algorithm performance, **c** shows relative importance of predictors, and **d** shows the calibration plot of the model

Model learning curve in Fig. 2a shows that increasing the number of learning examples (number of available visits) leads to a stable increase in the model performance indicating that the model does not suffer from major overfitting or representativeness problems. Increasing the number of available visit data for model training could even further improve the predictive performance.

In total, 25 variables were used for modeling. Dose percentage change was the most important predictor of a flare, followed by the DAS-28 ESR score, ESR, disease duration, CRP, and the duration of remission at study entry (Fig. 2c). Our model was very sensitive to missing data as depicted by a steep drop of model performance from an AUC of 0.8 to below 0.7 even with 5% missing data (Fig. 2b). Finally, our best fitting model in general tended to overestimate the risk of flare by as much as an absolute 30% especially in the low to mid-ranges of flare probability (Fig. 2d) [13].

## Discussion

In this study, we show that ML could be a reasonable approach to assess the individual flare risk in RA patients tapering anti-rheumatic treatment when reaching remission. The stacking meta-classifier method we used in this study provided a promising overall AUC of 0.81. The model learning curve in Fig. 2a suggests that our modeling approach still has room for improvement if it is trained on a larger dataset. Such approach could be further developed in the way that it assists decision-making with respect to treatment tapering with the aim to be more accurate in tapering and thereby reducing the incidence of flares and costs related to treatment.

The evaluation of this machine learning study is based on the high-quality data from the randomized-controlled RETRO trial. In contrast to previous evaluations performed as part of this study program, which were primarily dedicated to determining flare incidence at a cohort level and the effect of the intervention using conventional statistical techniques, this work focused on the individual flare probability and predictors at the individual level using an innovative machine learning approach. An advantage of this approach is that it is builds on basic predictors available in the usual clinical setting. As such, this ML-based predictive approach could tailor treatment tapering to the right patients thereby reducing the risk of flares but also the risk of unnecessary therapy.

There are several different learning methods that can create decision boundaries between classes (in our case flare yes vs. flare no). In order to select the best one, we have implemented a model selection approach based on nested cross-validation. It compared performance of logistic regression, k-nearest neighbors, naïve Bayes classifier, random forest and stacking meta-classifier. Our approach made it possible to compare optimal versions of these models as their corresponding hyper-parameters were continuously optimized within the inner loop of nested cross-validation. This approach is (1) extendable to yet more learning methods and (2) generalizable across different tasks and fields.

The current knowledge about the reliability and generalizability of ML approaches for predicting RA flares is very limited. One study showed that ML could be helpful to assess flares using data from electronic medical records (EMR) of RA patients [14]. EMR data typically are larger in size than data collected in clinical studies but are of lower quality, which may have hampered model training in this project. To our knowledge, there is no published work on developing such a model (also considering advanced ML models) from a high-quality, consistent dataset with minimal missing data collected in a clinical trial.

One particular concern with prediction models, be it ML or statistical, is overfitting [15]; especially when datasets and event numbers are relatively small. A meta-stacking classifier can create flexible new decision boundaries and improve classification performance by combining different decision characteristics of various classifiers. In our case, it improved AUC by four points compared to the best basic classification method (log. regression with the AUC of 0.76). Many solutions in data science competitions such as Kaggle (www.kaggle.com) apply similar ensemble learning methods. Since our best method tends to make the most out of existing data, it might as well be considered prone to overfitting. To avoid overfitting in our case, we used nested cross-validation [16]. This method splits training data for model fitting and validation before testing. Therefore, model performance estimates using nested cross-validation are rather conservative. Despite this conservative approach, our model reached a reasonable AUC of over 0.80.

It is known that certain characteristics of RA patients at the group level, such as autoantibody positivity are considered as risk factors for relapsing. Interestingly, these were not among high-ranking predictors in the individual model. Important predictors in this model were rather the drug dosage as well as clinical disease activity (DAS-28 ESR), disease duration, and inflammatory markers such as ESR or CRP. That dose reduction was the most important predictor of flares in our model is in line with the published RETRO results where 15.8% of the participants that continued treatment without change had relapsed while the relapse rate was 38.9% in the 50% dose reduction arm and 51.9% in the trial arm where treatment doses were reduced by 50% and subsequently stopped.

Some limitations of our study need to be underlined. We used a rather small sample of a relatively pure, complete, and consistent data set from a randomized controlled trial and the learning curves suggest that our model probably could not be trained to its full potential. Hence, the model calibration could be considered suboptimal; however, from a clinical standpoint, one may consider an overestimation of flare probability safer compared to an underestimation. Furthermore, probability calibration methods such as Platt’s method or isotonic regression can be used for further refinement. Although stacking meta-classifier provided the best accuracy for prediction as a point estimate, the confidence intervals around this suggests only the point estimate for the k-nearest-neighbor method as inferior from a frequentist perspective. To enable robust applicability of our modeling strategy in the future and implementation in the clinical care of patients with RA, it will be helpful to consider larger data sets and to test its use in a controlled clinical care setting.

Our study focused on patients under bDMARD therapy only and thus cannot make any conclusions for the tapering of conventional synthetic DMARDs. This approach was chosen because (i) tapering of bDMARDs is recommended before conventional synthetic (cs) DMARDs [17] and (ii) bDMARD treatment is rather costly as compared to csDMARDs. Finally, our prediction model was very sensitive to missing data and showed a considerable loss of predictive utility even when 5% of the predictor data was unavailable.

## Conclusions

Taken together, this is the first study showing that with a machine learning approach and high-quality data from a randomized controlled trial, it is possible to develop a model to predict the individual flare probability in RA patients in remission. Our modeling approach could be used to develop a clinical prediction tool for pilot implementation and prospective testing to further improve RA patient care.

## Supplementary Information

**Additional file 1.** Supplementary figures 1-8 and methods.

## Data Availability

All data generated or analyzed during this study are included in this published article.

## Abbreviations

ACPA: Anti-citrullinated protein antibodies
AUROC: Area under the receiver operating curve
bDMARDs: Biological disease-modifying anti-rheumatic drugs
CRP: C-reactive protein
csDMARDs: Conventional synthetic disease-modifying anti-rheumatic drugs
DAS-28 ESR: Disease Activity Score-28 based on erythrocyte sedimentation rate
HAQ: Health Assessment Questionnaire
ML: Machine learning
RA: Rheumatoid arthritis
RETRO: REduction of therapy in patients with rheumatoid arthritis in ongoing remission study
RF: Rheumatoid factor
tsDMARDs: Targeted-synthetic disease-modifying anti-rheumatic drugs
