# Supplementary material for: Advanced machine learning for predicting individual risk of flares in rheumatoid arthritis patients tapering biologic drugs
Source: Arthritis Res Ther. 2021 Feb 27;23:67. doi: 10.1186/s13075-021-02439-5 (PMC7913400; doi:10.1186/s13075-021-02439-5)
Supplement: Supplementary file 1 — Additional file 1. Supplementary figures 1-8 and methods. [file 13075_2021_2439_MOESM1_ESM.docx]

**Supplementary material to accompany**

Advanced Machine Learning for Predicting Individual Risk of Flares in Rheumatoid Arthritis Patients Tapering Biologic Drugs

Asmir Vodencarevic^1^*, Koray Tascilar^2,3^*, Fabian Hartmann^2,3^, Michaela Reiser^2,3^, Axel J. Hueber^2,3,4^, Judith Haschka^2,3,5^, Sara Bayat^2,3^, Timo Meinderink^2,3^, Johannes Knitza^2,3^, Larissa Mendez^2,3^, Melanie Hagen^2,3^, Gerhard Krönke^2,3^, Jürgen Rech^2,3^, Bernhard Manger^2,3^, Arnd Kleyer^2,3^, Marcus Zimmermann-Rittereiser^1^, Georg Schett^2,3^, David Simon^2,3^ on behalf of the RETRO study group

^1^Digital Health, Siemens Healthcare GmbH, 91052 Erlangen, Germany; ^2^Department of Internal Medicine 3 - Rheumatology and Immunology, Friedrich-Alexander University (FAU) Erlangen-Nürnberg and Universitätsklinikum Erlangen, 91054 Erlangen, Germany; ^3^Deutsches Zentrum fuer Immuntherapie (DZI), 91054 Erlangen, Germany; ^4^Section Rheumatology, Sozialstiftung Bamberg, 96049 Bamberg, Germany; ^5^St. Vincent Hospital, Vinforce Study Group, Medical University of Vienna, 1090 Vienna, Austria

**The RETRO trial**

RETRO was a multi-center, prospective, randomized, controlled, parallel-group trial that evaluated treatment withdrawal in rheumatoid arthritis (RA) patients in stable remission conducted between 2010 and 2018. Patients had to fulfil the 2010 ACR/EULAR criteria for the classification of RA for at least 1 year and had to be in remission, defined as a DAS-28 ESR score of less than 2.6 documented in 3 consecutive visits within the previous 6 months of screening under stable treatment with conventional synthetic DMARDs or biological DMARDs that were available at the time of study initiation (excluding Rituximab). Patients were randomized to 3 treatment arms. The control arm was followed without changing their baseline treatment. The reduce arm, was followed after all RA treatment was reduced by 50% at baseline and the reduce/stop arm in which all baseline treatments were reduced by 50% at baseline and stopped after 6 months. The total follow-up duration was 1 year with study visits conducted every 3 months. Primary endpoint of the study was the loss of DAS-28 remission, namely a DAS28-ESR value of more than 2.6. A flare was observed within 14 weeks for 23% of included 135 visits of the RETRO patients on biologics. This observed flare prevalence of 23% represents the flare risk on the cohort level that the individual flare risk should be compared against.

Fig. Supp. 1: Flowchart of the machine learning study


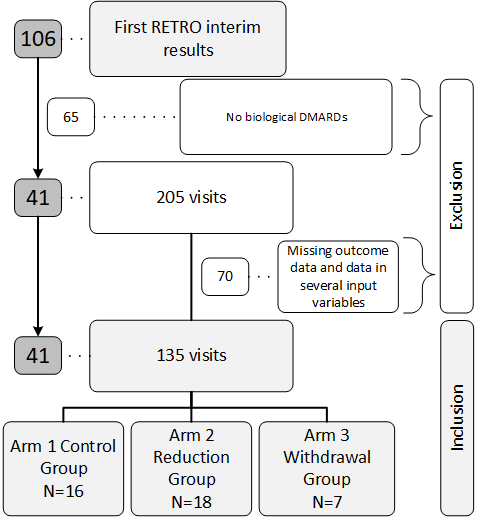


Fig. Supp. 2: An illustration of logistic regression based on the single input (predictor) variable.

| 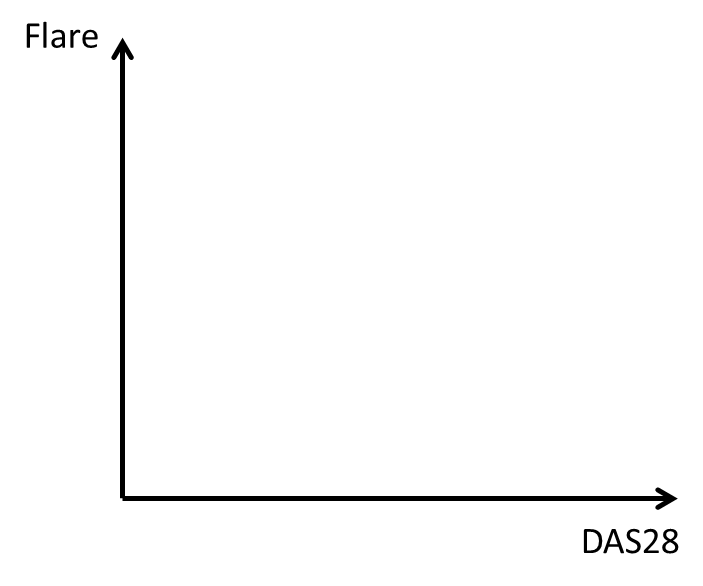   1. A single variable is used for classification | 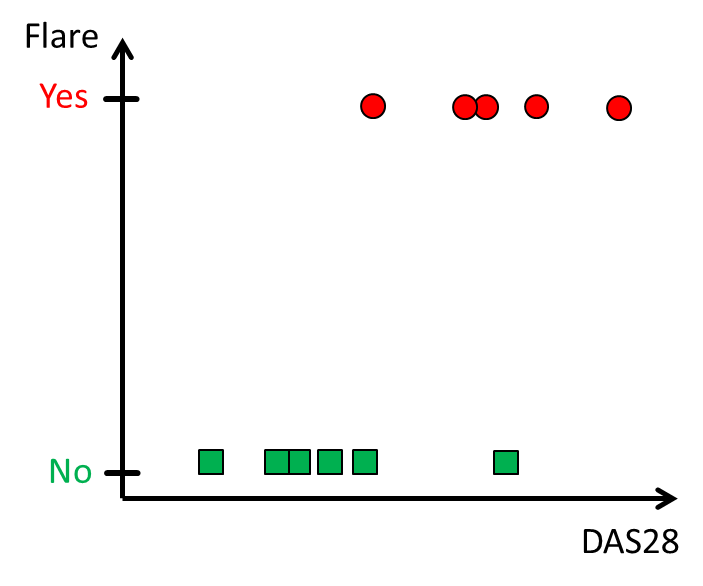   1. Training data points are plotted based on their class (factor level) (red: flare, green: no flare) |
| --- | --- |
| 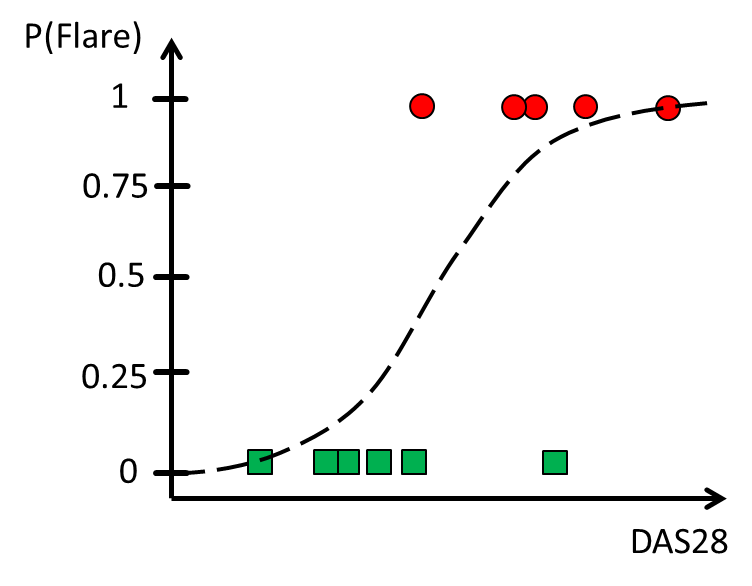   1. Logistic (sigmoid) function is fitted to training data | 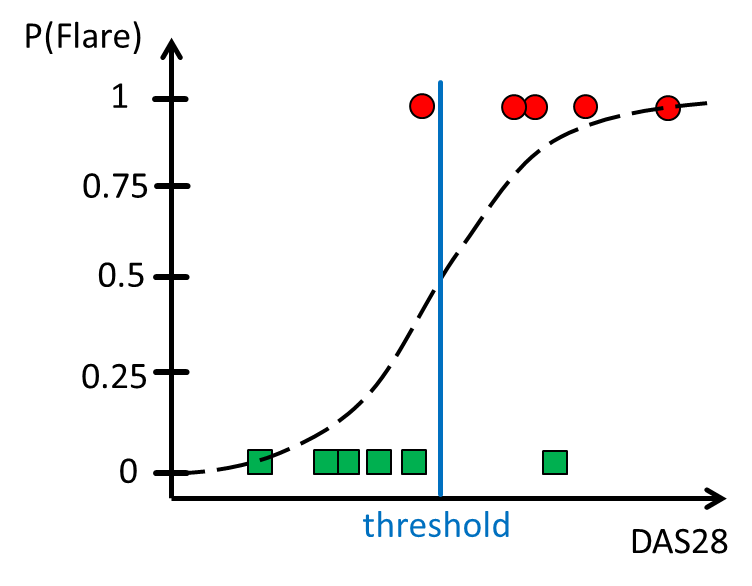   1. An optimal threshold is found to discriminate flares from no flares |
| 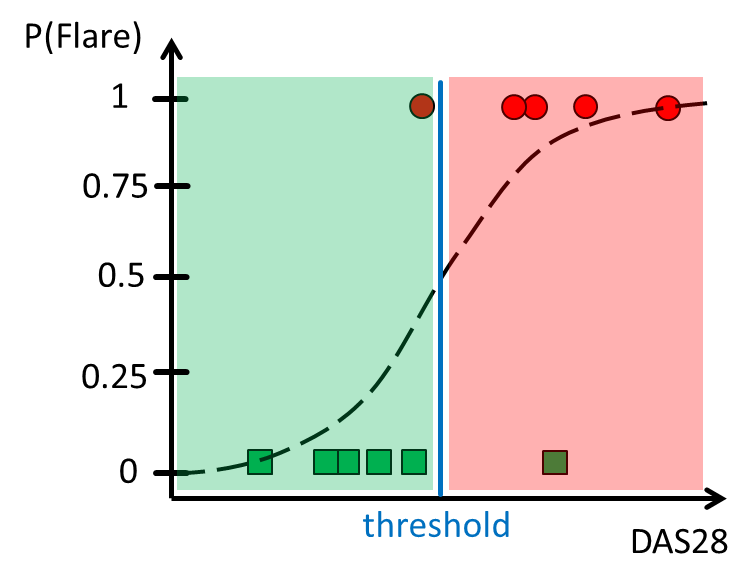   1. Whole range of DAS28 values is divided in two regions | 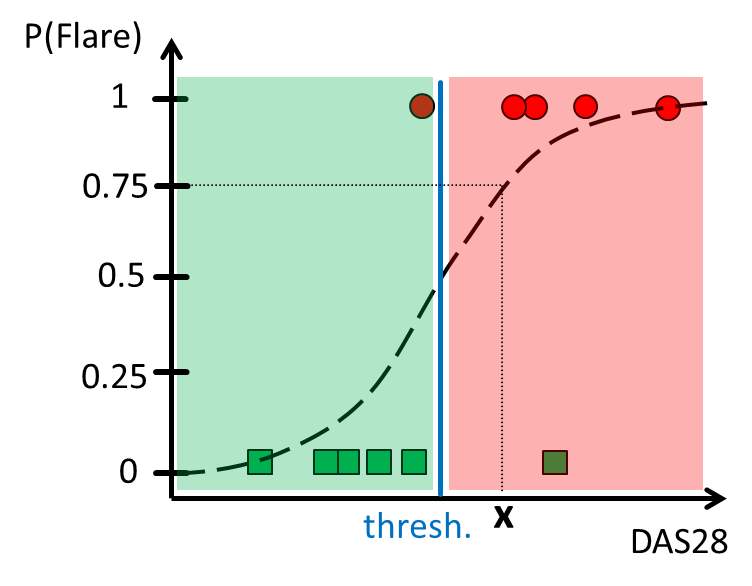   1. New instance **x** is classified based on the region it falls in (red with 75% probability) |

**Logistic regression**

Logistic regression is a well-known statistical model in which logarithm of the odds (log-odds) of the positive class (i.e. flare yes) is represented as a linear combination of the input variables. Fig. Supp. 2 illustrates how the method works in a simple example. In the first step the patient values are represented in the corresponding dimensionality of the input data (Fig. Supp. 2(b), only one input dimension, DAS-28 ESR shown for simplicity). In the next step, a logistic function is fitted to the data and it maps the values of DAS-28 ESR to the continuous interval [0,1], which represents flare probabilities (Fig. Supp. 2(c)). Then an optimal threshold is found which establishes a trade-off between false positives and false negatives (Fig. Supp. 2(d)) and the whole range of DAS-28 ESR values is divided into two regions based on this threshold (Fig. Supp. 2(e)). At this stage, the classifier is already built and can be applied to new instances. Fig. Supp. 2(f) shows how a new instance **x** is classified to the “flare yes” group based on its DAS-28 ESR value and the established threshold. Moreover, since this is a probabilistic classifier, the exact probability of the class “flare yes” can be computed using the sigmoid function. In this case, the probability of **x** belonging to the class “flare yes” is 75%.

Fig. Supp. 3: An illustration of k-nearest neighbors based on two input variables

| 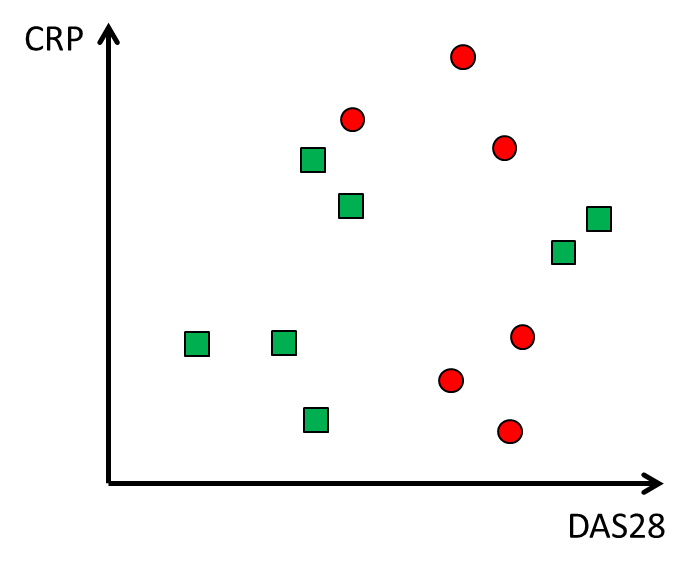   1. Training data is represented in two-dimensional input space   (red: flare, green: no flare) | 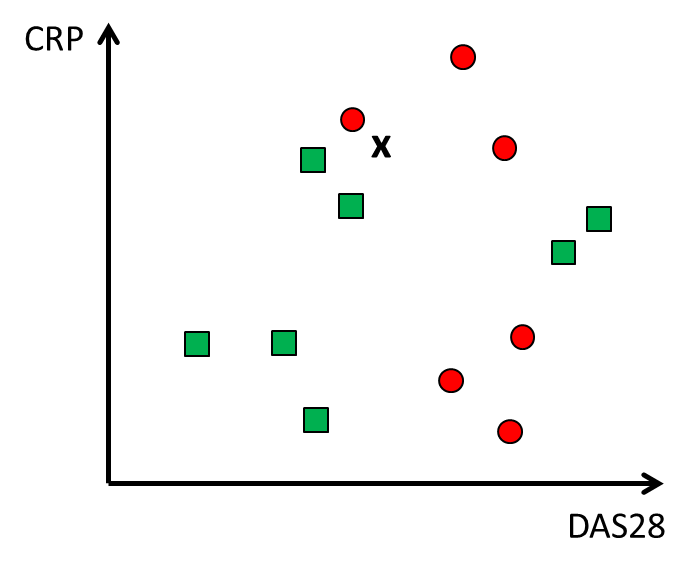   1. New instance **x** is added to the input space |
| --- | --- |
| 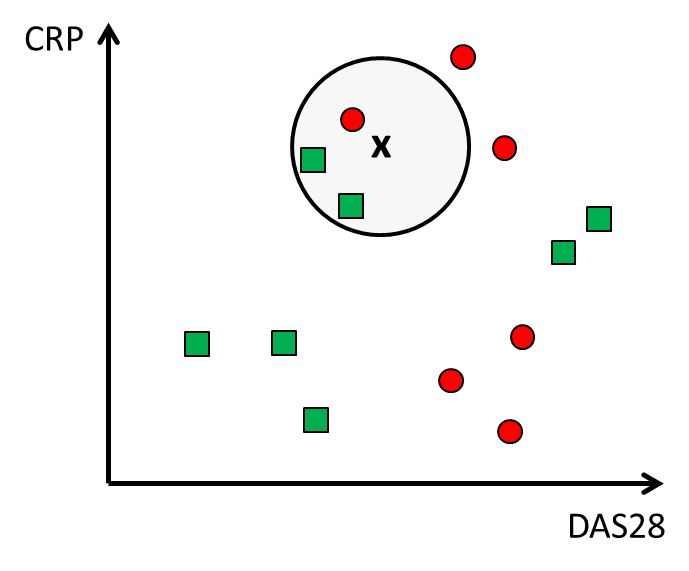   1. For k=3, the new instance is labelled as “green” with 67% probability | 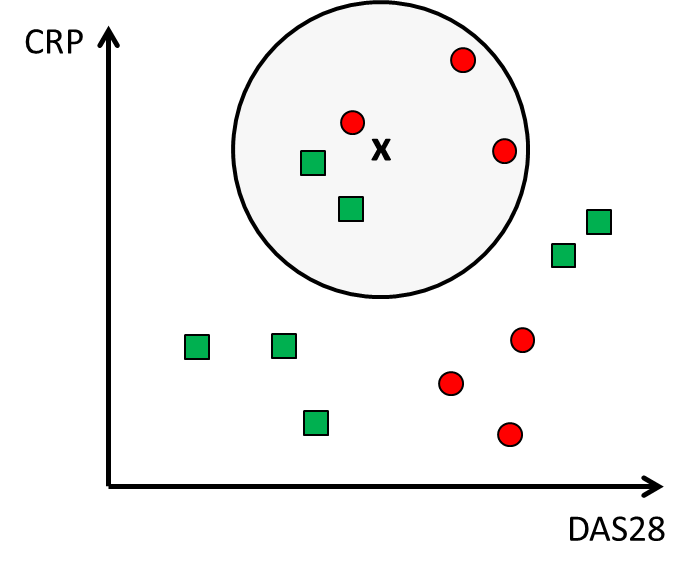   1. For k=5, the new instance is labelled as “red” with 60% probability |

**K-nearest neighbors**

K-nearest neighbors algorithm is one of the simplest models that classifies each new instance based on the majority class of its neighbors. Basically, the classifier only “memorizes” all the data and computes the distances between neighbors for each new instance that needs to be classified. The number of neighbors “k” is a parameter that needs to be adjusted to the specific problem (i.e no free lunch theorem). An illustration of this method is shown in Fig. Supp. 3. For simplicity, only two input dimensions are shown: C-Reactive Protein (CRP) and Disease Activity Score Erythrocyte Sedimentation Rate in 28 joints (DAS-28 ESR).

Fig. Supp. 4: An illustration of naïve Bayes classifier based on two input variables

| 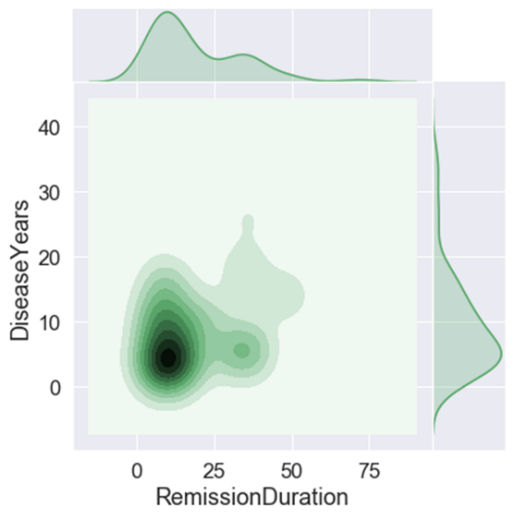   1. Independent conditional probabilities of two inputs given the class “flare no” and their product | 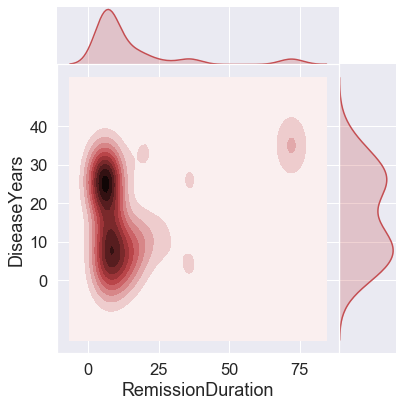   1. Independent conditional probabilities of two inputs given the class “flare yes” and their product |
| --- | --- |
| 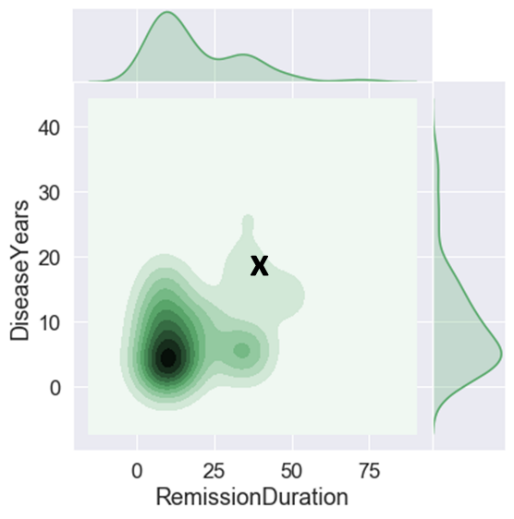   1. The posterior probability that **x** is “green” is measured | 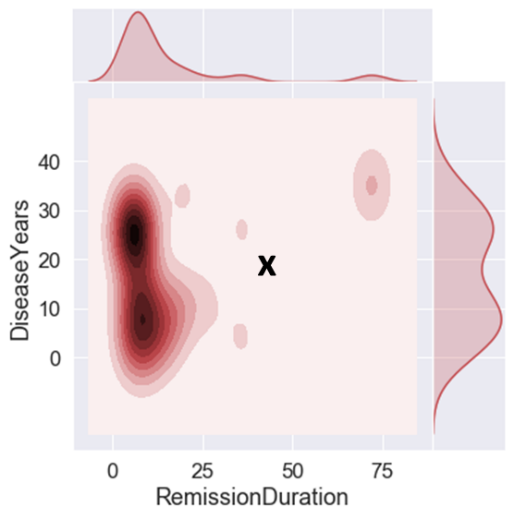   1. The posterior probability that **x** is “red” is measured |

Naïve Bayes classifier

Naïve Bayes is a probabilistic classifier which computes the posterior probability of flare based on Bayes theorem. The posterior probability is computed from the class prior probability and the likelihood (density function) that observed input values come from the given class. The main theoretical assumption of the naïve Bayes classifier is the independence of the input variables, which is rarely fulfilled in practice. Nevertheless, naïve Bayes performs well in many real-world problems. An algorithm illustration is given in Fig. Supp. 4 (again, only two variables are used for the sake of simplicity). Given classes “flare yes” (the “green” class) and “flare no” (the “red” class) the individual conditional probabilities are first computed for each input variable and then multiplied. This is shown in Fig. Supp. 4(a) and (b) for classes “flare yes” and “flare no” respectively. The new instance **x** is classified based on the Maximum A Posteriori (MAP) rule, i.e. as “flare no” (Fig. Supp. 4(c) and (d)) because $P\left( flare\_no | features \right)>P(flare\_yes|features)$; that is the probability of the new observation being a no flare given the predictors (its features) is higher than the probability of the observation being a flare.

Fig. Supp. 5: An illustration of how a decision tree is built. Random forests are ensembles of decision trees.

| 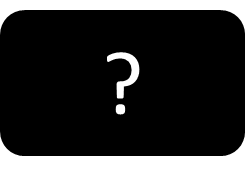   1. Which split minimizes impurity the most? | 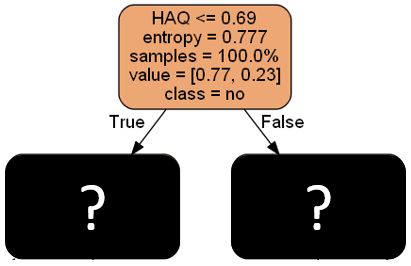   1. Highest impurity reduction when HAQ is split at value 0.69. |
| --- | --- |
| 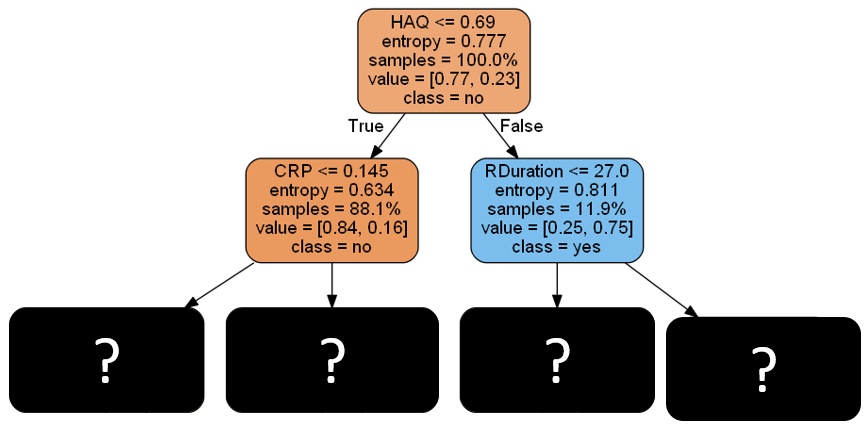   1. Next optimal splits are based on features CRP and RemissionDuration at their corresponding values. The algorithm recursively searches for the next optimal splits. | |

Random forest classifier

Random forest is an ensemble machine learning method based on decision trees. Multiple decision trees are trained on the original training dataset which is randomly sampled with replacement. For the training of each tree, only a randomly selected subset of available features (predictors) is used (e.g. square root of the total number of features). Each tree is created by evaluating which feature at which value should be used for splitting the dataset at each tree node in order to decrease “impurity” (also called entropy) of the resulting subsets. A feature which minimizes impurity is selected for splitting. In Fig. Supp. 5(a) this is the Health Assessment Questionary (HAQ) feature at the value 0.69. At newly obtained nodes, the procedure is repeated. After multiple decision trees are created (the number of trees is a parameter of random forest model), they classify new instances based on majority voting. E.g. if 150 out of 200 trees classify an instance as “flare yes”, then the final predicted label for the instance is “flare yes” with a probability of 75%.

Fig. Supp. 6: A schematic representation of the stacking meta-classifier


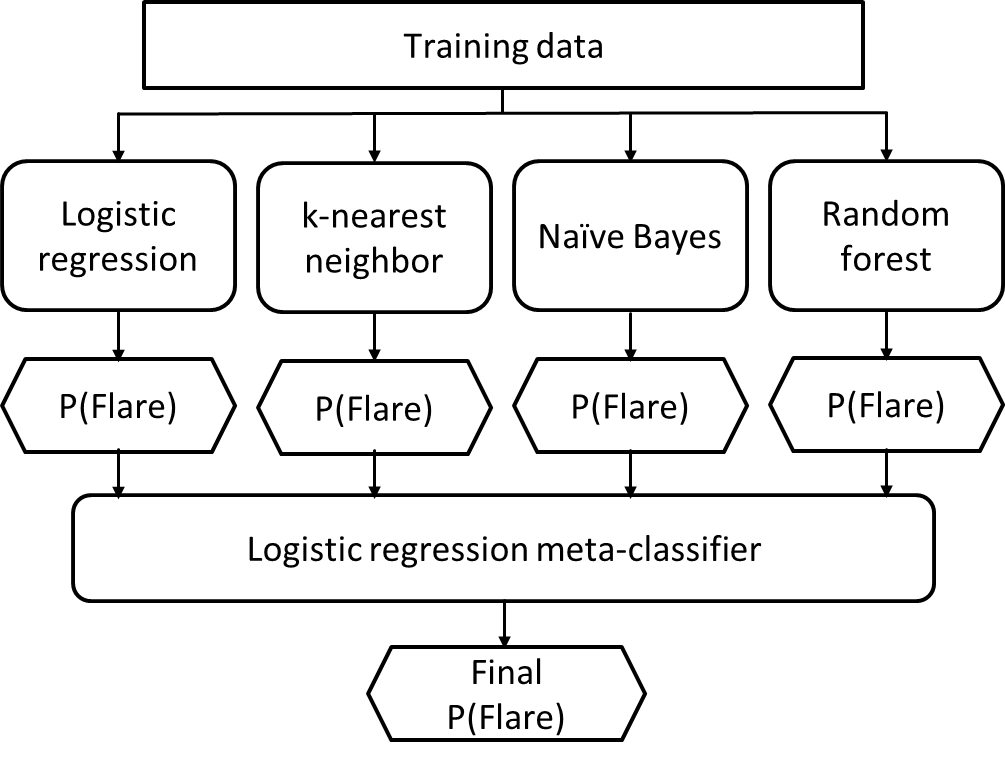


Stacking meta-classifier

Stacking is another ensemble learning technique that combines outputs (e.g. predicted probabilities) of multiple models into a single prediction using a meta-classifier. Unlike random forest which combines predictions coming from the same type of a model (i.e. decision tree), stacking can combine predictions of different types of models. As illustrated schematically in Fig. Supp. 6, stacking can include various “base” models like naïve Bayes and random forest whose outputs represent inputs to the logistic regression meta-classifier. Stacking can create very flexible decision boundaries between the classes and often provide higher classification accuracies. This is illustrated in Fig. Supp. 7.

| Fig. Supp. 7: An illustration of the flexible decision boundary obtained by stacking meta-classifier compared to other four models. For simplicity, only two input variables are used. |
| --- |
| 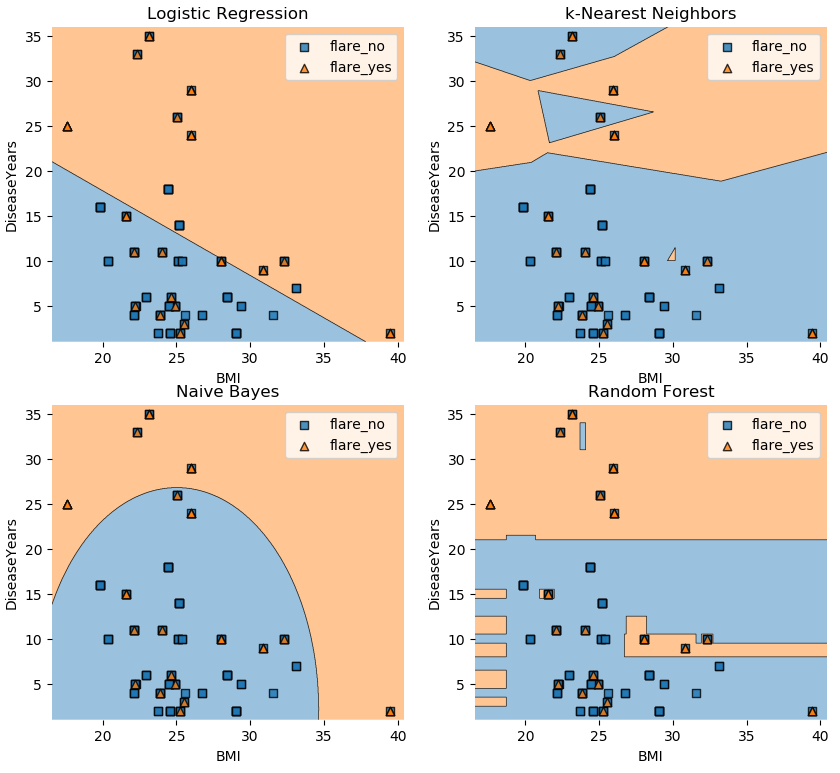   1. Decision boundaries of four base classifiers: linear for logistic regression, piecewise linear for k-nearest neighbors, (c) piecewise quadratic for naïve Bayes and piecewise linear parallel with axes for random forest |
| 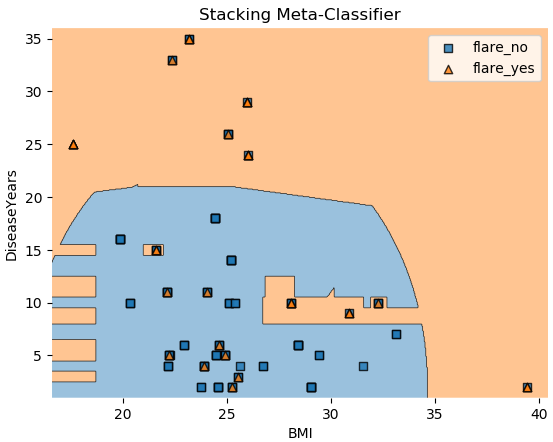   1. Flexible decision boundary of stacking meta-classifier obtained by combining decision boundaries of four base classifiers |

Fig. Supp. 8 Unbiased performance estimation, model training and optimization procedures

**Inputs:**

$DS$ dataset containing input variables *X* and output variable *y*

$K_{I}$ number of inner cross-validation folds # 3 in our implementation

$K_{O}$ number of outer cross-validation folds # 5 in our implementation

$I$= number of random search iterations # 9 in our implementation

$M=\{M_{LR}, M_{KNN}, M_{NB}, M_{RF}, M_{SMLR}\}$ set of untrained classifiers including base classifiers: logistic regression, k-nearest neighbors, naïve Bayes, random forest as well as

logistic regression stacking meta-classifier

$HP$ set of all combinations of predefined values for hyperparameters of models in $M$

**Outputs:**

$AUC$ unbiased performance estimate measured as the area under the ROC curve

$M_{final}$ final trained and optimized set of models $M$

**Procedure: Nested cross-validation with random search for unbiased performance estimation**

$$for i=1 to K_{O}:$$

Split $DS$ into ${DS}_{i}^{train}$ and ${DS}_{i}^{test}$

$$for j=1 to K_{I}:$$

Split ${DS}_{i}^{train}$ into ${DS}_{j}^{train}$ and ${DS}_{j}^{val}$

$$for k=1 to I:$$

$${hp}_{k}= RandomSelect(HP)$$

Create $M_{k}$ by training $M$ on ${DS}_{j}^{train}$ using ${hp}_{k}$

Compute validation performance ${AUC}_{j}^{val}$ of $M_{k}$ on ${DS}_{j}^{val}$

Select ${hp}_{i}$ from all randomly selected ${hp}_{k}$ which maximizes ${AUC}_{j}^{val}$

Create $M_{i}$ by training $M$ on ${DS}_{i}^{train}$ using ${hp}_{i}$

Compute test performance ${AUC}_{i}^{test}$ of $M_{i}$ on ${DS}_{i}^{test}$

Compute unbiased performance estimate as $AUC= \frac{1}{K_{O}}\sum_{i=1}^{K_{O}} {AUC}_{i}^{test}$

**Procedure: Training and optimization of final model**

$$for j=1 to K_{I}:$$

Split $DS$ into ${DS}_{j}^{train}$ and ${DS}_{j}^{val}$

$$for k=1 to I:$$

$${hp}_{k}= RandomSelect(HP)$$

Create $M_{k}$ by training $M$ on ${DS}_{j}^{train}$ using ${hp}_{k}$

Compute validation performance ${AUC}_{j}^{val}$ of $M_{k}$ on ${DS}_{j}^{val}$

Select ${hp}_{optimal}$ from all randomly selected ${hp}_{k}$ which maximizes ${AUC}_{j}^{val}$

Create $M_{final}$ by training $M$ on $DS$ using ${hp}_{optimal}$
